# *In Situ* Identification of Plant-Invasive Bacteria with MALDI-TOF Mass Spectrometry

**DOI:** 10.1371/journal.pone.0037189

**Published:** 2012-05-17

**Authors:** Dominik Ziegler, Anna Mariotti, Valentin Pflüger, Maged Saad, Guido Vogel, Mauro Tonolla, Xavier Perret

**Affiliations:** 1 Department of Botany and Plant Biology, University of Geneva, Geneva, Switzerland; 2 Institute of Microbiology, Bellinzona, Switzerland; 3 Mabritec AG, Riehen, Switzerland; The George Washington University, United States of America

## Abstract

Rhizobia form a disparate collection of soil bacteria capable of reducing atmospheric nitrogen in symbiosis with legumes. The study of rhizobial populations in nature involves the collection of large numbers of nodules found on roots or stems of legumes, and the subsequent typing of nodule bacteria. To avoid the time-consuming steps of isolating and cultivating nodule bacteria prior to genotyping, a protocol of strain identification based on the comparison of MALDI-TOF MS spectra was established. In this procedure, plant nodules were considered as natural bioreactors that amplify clonal populations of nitrogen-fixing bacteroids. Following a simple isolation procedure, bacteroids were fingerprinted by analysing biomarker cellular proteins of 3 to 13 kDa using Matrix Assisted Laser Desorption/Ionization Time of Flight (MALDI-TOF) mass spectrometry. In total, bacteroids of more than 1,200 nodules collected from roots of three legumes of the *Phaseoleae* tribe (cowpea, soybean or siratro) were examined. Plants were inoculated with pure cultures of a slow-growing *Bradyrhizobium japonicum* strain G49, or either of two closely related and fast-growing *Sinorhizobium fredii* strains NGR234 and USDA257, or with mixed inoculants. In the fully automatic mode, correct identification of bacteroids was obtained for >97% of the nodules, and reached 100% with a minimal manual input in processing of spectra. These results showed that MALDI-TOF MS is a powerful tool for the identification of intracellular bacteria taken directly from plant tissues.

## Introduction

Of the diverse microorganisms that thrive in the rhizosphere of higher plants, only a few can colonize the inner root tissues. Fewer still can enter plant cells and establish persistent intracellular infections. Amongst them, rhizobia form a heterogeneous group of α- and β-proteobacteria capable of establishing beneficial nitrogen-fixing associations with legumes [1]. Rhizobia are responsible for as much as half of the nitrogen fixed each year by terrestrial biological systems [2], and are often used in agriculture to supplement plants with the reduced forms of nitrogen absent in many soils around the world. Reduction of atmospheric nitrogen (N_2_) by rhizobia occurs predominantly inside plant cells found in specialized root (occasionally stem) organs called nodules. To colonize the inner tissues of nodules, rhizobia generally follow a trans-cellular path of infection that crosses the root epidermis as well as several layers of cortical cells. This infection process remains mostly under the control of host plants and rhizobial proliferation is restricted to the tips of infection threads (ITs), which ultimately ramify when reaching the newly formed nodule meristem [3], [4]. Plants restrict detrimental infections of root tissues by harmful pathogens or poor nitrogen fixers by using sophisticated screens that involve the coordinated exchange of molecular signals between nodulating rhizobia and host plants [5], [6]. Flavonoids released by roots, bacterial surface polysaccharides, nodulation factors (Nod-factors) and type-three secreted proteins released by rhizobia are amongst the most discriminating signals involved in the establishment of a proficient symbiosis. Ultimately, rhizobia are released from ITs into the cytoplasm of nodule cells where they differentiate into nitrogen-fixing bacteroids. In return for reduced nitrogen, plants provide bacteroids with ample carbon supply mostly in the form of dicarboxylates. To protect rhizobial nitrogenase from its irreversible inactivation by traces of free oxygen, plants have evolved a number of mechanisms including a cortical oxygen diffusion barrier that surrounds the infected nodule tissue and the synthesis of leghemoglobin, which together regulate the oxygen tension within infected cells. Leghemoglobin is particularly abundant inside N_2_-fixing nodules where it accounts for as much as 30% of all proteins [7]. As a single rhizobial cell attached at the root-hair tip is sufficient to initiate the formation of an infection thread, a population of bacteroids inside a nodule is mostly clonal. In fact nodules can be regarded as small bioreactors in which each of the infected nodule cells contains several hundred bacteroids. Occasionally infection threads can accommodate distinct bacteria, and thus may lead to nodules that house a mixed population of rhizobia [3], [4].

**Figure 1:**
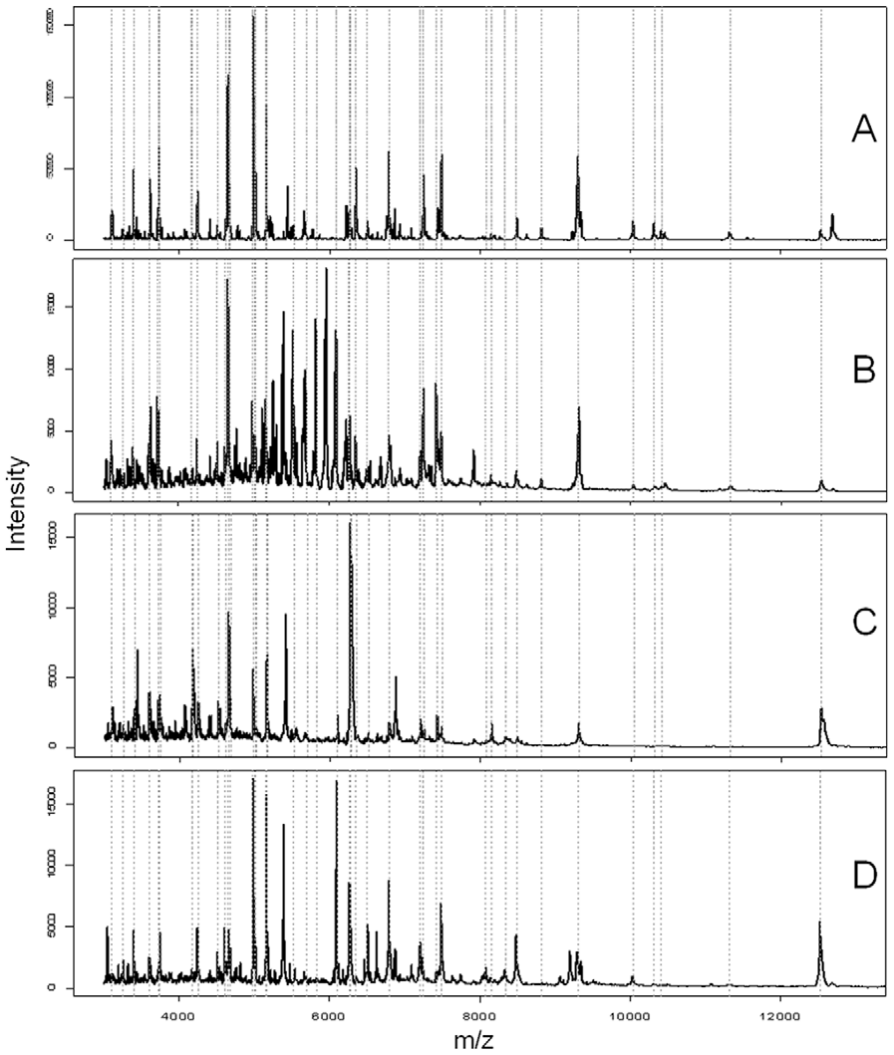
Typical MALDI mass spectra between *m/z* 3000 and *m/z* 13000 of *S. fredii* strain USDA257 grown on TY for 5 days at 28°C (A) or found inside nitrogen-fixing nodules of *G. max* (B), *M. atropurpureum* (C), and *V. unguiculata* (D) collected respectively 35, 49, and 42 days post-inoculation. Sample preparation included washing steps to remove leghemoglobin, and suspension of bacterial pellets in 25% formic acid. Positions of biomarkers selected for the USDA257 SSp are showed with dashed lines.

Matrix-assisted laser desorption/ionization time of flight (MALDI-TOF) mass spectrometry (MS) has become a method of choice for the identification of bacteria [8]. In such procedures, isolated bacteria are first grown as micro-colonies on solid media prior to MALDI-TOF MS analysis. Then, small numbers of cells are lysed to release the intracellular proteins, which in turn will be ionized and separated according to their mass to charge ratio (*m/z*). Protein masses are recorded as distinct peaks that together form a complex spectrum or fingerprint, which is characteristic of a bacterial sample (see Fig. 1). Each spectrum of protein masses is subsequently matched against reference spectra that are stored in dedicated databases, and were initially established using well characterized reference strains and defined growth conditions [9]. The Spectral Archive and Microbial Identification System (SARAMIS™ of database management reduces the complexity of mass spectra by restricting datasets to subsets of peaks from the most abundant proteins or peptides found in the 2 to 20 kDa range. These selected peaks together form the so called SuperSpectra™ (SSp) that predominantly include ribosomal proteins as selected biomarkers [10]. Depending on the number of SSp that are stored in a reference database for a given group of bacteria, a sampled strain can be reliably assigned to a known family, genus, species or more rarely a particular subspecies [11], [12], [13], [14], [15].

Except for a few protocols in which infectious bacteria from urine or blood samples were concentrated by centrifugation prior to cell typing [16], [17], [18], the identification of environmental or pathogenic bacteria by MALDI-TOF MS analysis requires preliminary steps of bacterial isolation and purification. Unlike most animal pathogens that form isolated micro-colonies in a matter of hours, plant-interacting bacteria require longer incubation times that, as in the case of bradyrhizobia, may extend over several days. To prevent growth of soil contaminants during such long incubation periods, standard methods for the identification of endosymbiotic rhizobia include a surface-sterilization of root nodules prior to sampling serial dilutions of their contents on plates [19]. Recently, MALDI-TOF MS technology was applied to the identification of fast–growing rhizobia, but only to strains cultivated on solid media [20]. To bypass the initial and time-consuming steps of bacterial isolation and subsequent cultivation, we explored the possibility of applying MALDI-TOF MS analysis directly to populations of endosymbiotic bacteroids found in root nodules. For this study, three symbiotic strains were used in combination with the three host plants: *Vigna unguiculata* (cowpea), *Macroptilium atropurpureum* (siratro) and *Glycine max* (soybean). With a generation time close to 3 h., the fast-growing *Sinorhizobium fredii* strain NGR234 was selected for its unique ability to induce nodule formation on more than 120 genera of legumes, and fix nitrogen with a subset of at least 150 plants [21]. The other fast-grower *S. fredii* strain USDA257 shares with NGR234 many of its symbiotic genes [22], [23], but unlike NGR234 forms N_2_-fixing symbioses with selected soybean cultivars [21]. In contrast, the slow-growing micro-symbiont *Bradyrhizobium japonicum* strain G49 is genetically distant from NGR234 and USDA257 and fixes nitrogen in association with diverse legumes, including *G. max*, *M. atropurpureum* and *V. unguiculata*. Using these distinct strains, we show here that MALDI-TOF MS can allow for the rapid and reliable identification of nodule bacteria, including at the subspecies level, thus paving the way for large-scale population studies of rhizobia isolated from root nodules found on crops of agronomical importance.

## Results

### MALDI-TOF MS fingerprinting of free-living rhizobia

Reliable identification of bacterial strains requires the selection as biomarkers of protein masses that appear consistently in spectra, irrespective of the growth conditions or age of the cultures. Thus, cells of strains G49, NGR234, and USDA257 were grown at 28°C on solid media for 2, 3, 5, 7, 10, and 14 days. Initially, bacterial samples were subsequently prepared using either (i) the direct smear method, (ii) a suspension in 25% formic acid, or (iii) a pre-purification step for cellular proteins via an extraction with acetonitrile and 70% formic acid. For each strain, 18 averaged spectra of good quality (generated using the 3 methods of sample preparation for each of the 6 incubation periods) were compiled into a single representative SuperSpectrum™ (SSp) comprised of 39 to 42 biomarker masses that are listed in Table S1. Because of their abundance, small size and ubiquitous distribution, ribosomal proteins are often selected as biomarkers [10], [24] that, in some cases, are sufficiently discriminatory to allow for an accurate typing at the strain level [25], [26]. Accordingly, 35 of the 108 biomarkers selected for the SSp of G49, NGR234 and USDA257 were tentatively assigned to ribosomal proteins with corresponding molecular weights that were calculated using the corresponding genomic sequences (see Table S1). As befits strains that belong to the same *Sinorhizobium* genus, the reference spectra of NGR234 and USDA257 shared 12 biomarkers of which 7 were identified as ribosomal proteins. In contrast, none of the 39 biomarkers specific to G49 were found in NGR234 or USDA257, and none of the 108 masses listed in Table S1 were common to all three rhizobial SSp. Ultimately, the sets of biomarker masses were matched against each other and against the whole SARAMIS database (ver. 4.09, system ver. 3.4.1.2, with >70,000 spectra and >2,600 SSp). This procedure showed that an unequivocal strain assignment was reached when at least 60% of the biomarkers of a given spectrum of G49, NGR234 or USDA257 matched those of the reference SSp. Thus, the identification thresholds were set at 24 (from a total of 39), 25 (of 40) and 26 (of 42) matching biomarkers for the spectra of respectively, strains G49, NGR234 and USDA257.

### Generating reproducible spectra of root-nodule bacteria

**Figure 2:**
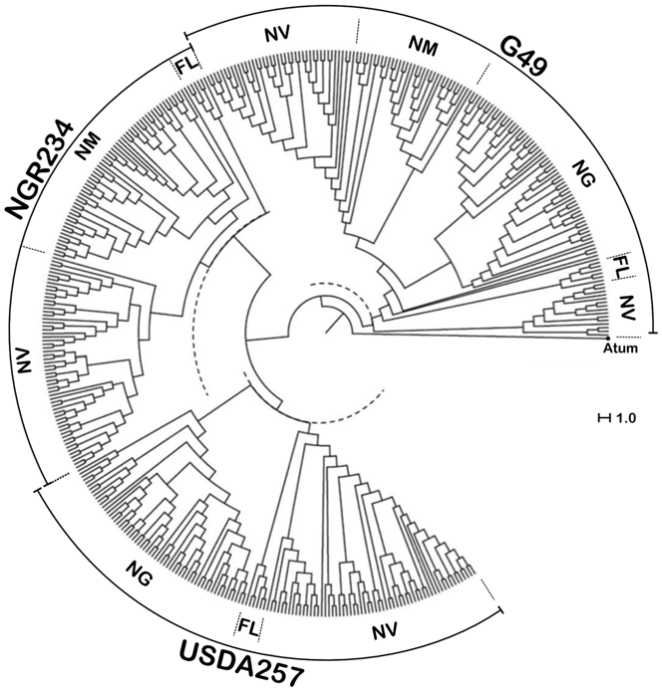
Unsupervised hierarchical cluster analysis (Dice algorithm) of G49, NGR234 and USDA257 MALDI-TOF MS mass spectra comprised in the size range of *m/z* 3000 to 15000. Bacteria were isolated from free-living cultures (FL), or nodules of *G. max* (NG), *M. atropurpureum* (NM), or *V. unguiculata* (NV). The respective positions of the 60% identity level used as a threshold to assign strains are shown as dotted semi-circles. *Agrobacterium tumefaciens* (Atum) was selected as the outgroup.

To establish a reliable protocol of rhizobial identification that was both robust and able to be applied to many symbioses, a number of plant-microsymbiont combinations were tested. Batches of 16 plants of *G. max* cv. Davis, *M. atropurpureum* (DC.) Urb., or *V. unguiculata* cv. Red Caloona were inoculated with either strain G49, NGR234 or USDA257, corresponding to a total of 144 plants in 72 Magenta jars for each legume species. Except for NGR234 that forms uninfected pseudonodules on *G. max* and USDA257 that fixes poorly with *M. atropurpureum*, all remaining plant-bacterium combinations lead to proficient nitrogen-fixing associations. To avoid potential problems linked to senescence of bacteroids, nitrogen-fixing nodules of cowpea, siratro, and soybean were harvested before flowering time at respectively, 42, 49 and 35 days post-inoculation (dpi) and stored at −60°C until further use. Following surface sterilisation, nodules were processed as described in the materials and methods section to remove contaminating leghemoglobin from the samples. Enriched bacteroid pellets were subsequently treated using either of the three methods described above for free-living bacteria, and two replicates for each nodule. A total of 420 nodules were examined in this way, including 60 nodules for each of the following microsymbiont/host-plant combinations G49/cowpea, G49/soybean, NGR234/cowpea, NGR234/siratro, USDA257/cowpea and USDA257/soybean, and 30 nodules for the G49/siratro and USDA257/siratro combinations. Representative spectra of free-living or endosymbiotic cells of USDA257 are shown in Figure 1. Using the threshold of 60% biomarkers in common that was established earlier for G49, NGR234 or USDA257 SSp, a positive strain-identification was reached for 389 of the 420 nodules (92%). When the 30 small and poorly infected nodules formed by USDA257 on siratro were excluded from the analysis, the success rate for the identification of nodule bacteria increased to 97% (379 out of 390 nodules). However, nodule bacteria from the remaining 11 unassigned nodules (3%) were identified by matching manually each of the unknown spectra against the representative spectra selected to generate the SSp of G49, NGR234 or USDA257. Thus, with a minimal operator input, bacteroids from all nodules were successfully identified. The circular dendogram presented in Figure 2 shows the unambiguous clustering in distinct G49, NGR234 or USDA257 groups of 410 individual spectra obtained from free-living bacteria or nitrogen-fixing bacteroids, using *Agrobacterium tumefaciens* as an outgroup. Ultimately, as spectra of optimal quality were produced using the preliminary treatment of isolated bacteria in 25% formic acid, all subsequent samples were prepared in this way.

### A competition assay using MALDI-TOF MS to identify nodule bacteria

In the soil, competition between rhizobia to gain access to the nodule environment is widespread and often limits attempts to use improved strains for field inoculations [27], [28]. To assess the reliability of the MALDI-TOF MS protocol for identifying root-nodule bacteria, a competition assay was undertaken. Cowpea was selected as a host plant because of its low selectivity for rhizobia [29]. Sinorhizobia NGR234 (Rif^R^, Km^S^) and USDA257 (Rif^S^, Km^R^) were chosen as microsymbionts because both strains (i) are genetically closely related, (ii) have orverlapping host-ranges [21], and (iii) possess distinct antibiotic resistances that facilitate their identification using appropriate selective media. Inoculants of 2×10^8^ rhizobia per plant were prepared using mixtures of NGR234 and USDA257 cells at ratios of 100/1, 50/50 or 1/100. Plants were harvested 42 dpi and a total of 113 to 120 nodules from up to 11 plants were examined for each of the three inoculants (Table 1). Nodule occupancy was determined using both MALDI-TOF MS spectra of bacteroids and by plating isolated nodule bacteria on selective media. In all conditions tested, NGR234 outcompeted USDA257 for nodulation on *V. unguiculata* including when cells of NGR234 were outnumbered by a factor of 100. Although MALDI-TOF MS analysis systematically resulted in the identification of a single nodule strain (most probably the strain with the most abundant bacteroid population), growth on selective media confirmed that 4 to 25% of the nodules were infected with both NGR234 and USDA257. As no bacterial growth was observed on plates on which surface-sterilized nodules were rolled, the sterilization procedure was efficient and cases of nodule occupancy documented as mixed were unlikely to result from contaminating bacteria found on the nodule epidermis. In a similar assay using G49 and NGR234 as competitors, the analysis of an additional 484 nodules showed that G49 outcompeted NGR234 for nodulation on *V. unguiculata* irrespective of the cell ratios used as inoculants (data not shown). These competition assays also showed that a correct identification of the predominant bacteroid population was reached by SARAMIS™ in the automatic mode for 753 (90%) of the 837 nodules examined, while strain identification for the remaining 84 nodules required only a minimal operator input.

**Table 1:** Identification of bacteria competing for nodulation on *V. unguiculata*.

		NGR234 vs. USDA257		
	Cell ratio	100/1	50/50	1/100
	Nodules/plants analyzed	120/10	120/11	113/11
Nodule occupancy	NGR234	115 (95.9)	102 (85.0)	64 (56.6)
	USDA257	0 (0.0)	5 (4.2)	20 (17.7)
	mixed – NGR234 spectra	4 (3.3)	4 (3.3)	5 (4.4)
	mixed – USDA257 spectra	1 (0.8)	9 (7.5)	24 (21.2)
Numbers of manual inputs in MALDI analysis		11 (9.2)	3 (2.5)	2 (1.8)

% of total nodules are shown in brackets.

## Discussion

That NGR234 so efficiently outcompeted USDA257 for nodulation on *V. unguiculata* was surprising since both strains are genetically closely related, secrete Nod-factors of similar structures, and have largely overlapping host-ranges [6]. The absence of functional copies of the *nodSU* genes [30] is probably not the reason for the lower competitiveness of USDA257, as a mutant of NGR234 deleted in *nodSU* nodulates cowpea with the same efficiency as the wild type [31]. It seems likely that competitiveness of NGR234 and USDA257 on *V. unguiculata* is not mediated by Nod-factors, but rather due to specific alterations in the composition and structure of their respective cell surface components. Both NGR234 and USDA257 activate type three protein secretion systems (T3SS) in response to flavonoid stimulation [32], yet the presence or absence of a functional T3SS has no measurable effect on the nodulation of cowpea by NGR234 [33]. Instead, flavonoid-inducible modifications in the *O*-antigen portion of lipopolysaccharides were shown to affect the ability of NGR234 to fix nitrogen on *V. unguiculata* [34]. As genes responsible for rhamnan synthesis are absent from USDA257 [23], surface polysaccharides are candidate determinants of the NGR234 competitivity on cowpea.

**Figure 3:**
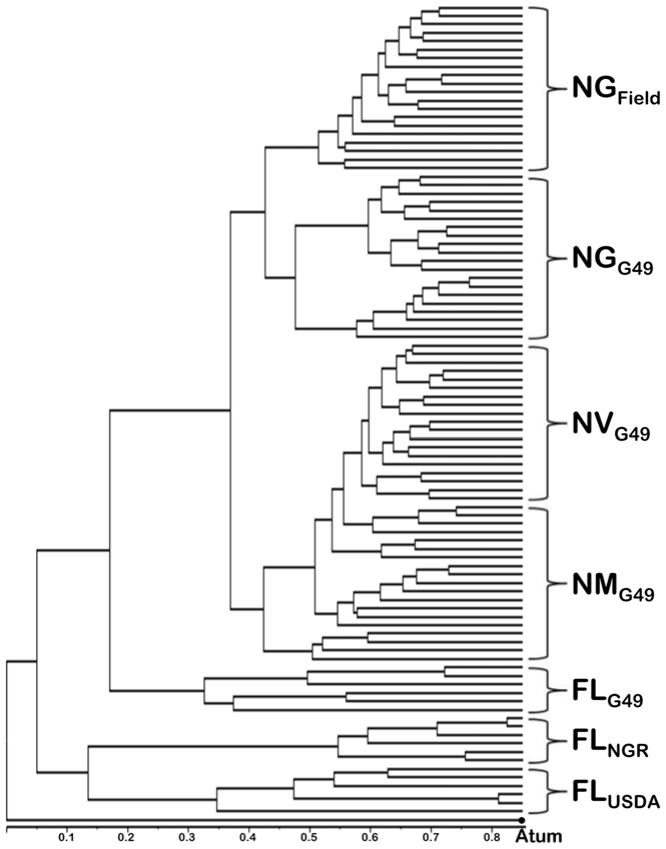
Typing of bacteroids found in nodules of a soybean field. Unsupervised hierarchical cluster analysis (Dice algorithm) of complex MALDI-TOF MS spectra (*m/z* 3000 to 15000) of rhizobia found inside nodules of *G. max* from a field in the Swiss state of Ticino (NG_Field_), or of G49 isolated from nodules of soybean (NG_G49_), siratro (NM_G49_), and cowpea (NV_G49_). Spectra from free-living cells of G49 (FL_G49_), NGR234 (FL_NGR_), and USDA257 (FL_USDA_), were also included. *A. tumefaciens* (Atum) was used as an outgroup.

More importantly, the competition assays confirmed that our protocol for bacteroid identification via MALDI-TOF MS was capable of discriminating between closely related strains such as NGR234 and USDA257. The possibility to bypass the time-consuming step of cultivating individual colonies on plates to reach a reliable identification of nodule bacteria by MALDI-TOF MS opens up new perspectives. According to our data, bacteroids found within a single mature nitrogen-fixing nodule are in sufficient numbers to generate reproducible spectra of good quality. Except for the abundant leghemoglobin that needs to be washed away to prevent the saturation of spectra, nodule cells or their proteins do not seem to interfere with the process of bacteroid identification. Although spectra of USDA257 isolated from nodules of distinct plants appeared to differ (see Fig. 1), a correct identification was ultimately reached because only the presence or absence (and not the respective signal intensities) of specific biomarker masses were used as discriminating quanta. That one SSp generated from spectra of free-living rhizobia was sufficient to reach a correct strain identification, irrespective of the plant from which the bacteroids were collected, facilitates further developments. The need to improve the SARAMIS database with SSp's of both free-living and endosymbiotic rhizobia would have been impractical, given that some promiscuous strains may nodulate more than one hundred genera of legumes [21]. Currently, a rhizobia-specific database is being established using well-characterized fast- and slow-growing strains belonging to the α- and ß-proteobacteria. Once established, such a database is bound to facilitate the implementation of MALDI-TOF MS as a tool to study the great diversity in populations of symbiotic rhizobia in agricultural or natural soils. To test whether our protocol for strain identification was efficient for such a purpose, 24 nodules were collected in a field of mature soybeans in the Swiss state of Ticino. MALDI-TOF MS spectra of the bacteroids found within these nodules were matched against those of cells of G49, NGR234 and USDA257 isolated from nodules of plants grown in laboratory conditions. Figure 3 shows that field-nodule bacteria clustered together with G49 bacteroids isolated from nodules of *G. max* cv. Davis while free-living cells and bacteroids of G49 found in cowpea or siratro formed distinct subclusters. This result is consistent with the use of *B. japonicum* strains as commercial inoculants for soybean cultures (including in Switzerland), and confirms that direct identification via MALDI-TOF MS of strains found in environmental samples is possible.

However, nodules come in many forms and shapes in nature, and the maturation of bacteroids and their fate once nodules senesce vary considerably in a host-dependent manner [1]. For example, nodules of cowpea, siratro and soybean, are all of determinate type with populations of bacteroids being regarded as relatively homogenous and to some extent capable of resuming growth once outside of plant cells [35], [36]. In contrast, nodules of the indeterminate type are characterized by the presence of a persistent meristem, and several developmental zones within which bacteroids display distinct differentiation states [37]. Legumes of the inverted repeat-lacking clade (IRLC) such as *Medicago sativa*, form indeterminate nodules in which nitrogen-fixing bacteroids are terminally differentiated [38]. Terminal differentiation results from the action of abundant nodule-specific and cystein-rich peptides (NCRs) of 3 to 5 kDa that induce the endoreduplication of the bacteroid genome and a concomitant cell enlargement [39], [40]. It is unclear whether the endoreduplicated state of *Sinorhizobium meliloti* bacteroids in indeterminate nodules of *M. sativa* may affect the process of strain identification by MALDI-TOF MS. In this respect, further studies will also confirm whether NCRs that are ultimately targeted to bacteroids, can be identified by comparing the spectra of free-living and endosymbiotic cells of *S. meliloti*. Nevertheless, our current protocol of strain identification suits many agronomically important legume crops such as soybean, bean (*Phaseolus vulgaris*) or pigeon pea (*Cajanus cajan*), all of which develop determinate nodules on their roots.

## Materials and Methods

### Characteristics of bacteria and growth conditions

Derivatives of *S. fredii* strains NGR234 resistant to rifampicin (Rif^R^) [41] and USDA257 resistant to kanamycin (Km^R^) [42] were grown at 27°C in/on TY [43], supplemented with antibiotics at final concentrations of 50 µg ml^−1^. *B. japonicum* strain G49 was isolated from a batch of the commercial inoculum HiStick Soybean (Becker Underwood, Littlehampton, West Sussex, UK) and grown in/on yeast-mannitol (YM) medium. Strain G49 was sensitive to both, rifampicin and kanamycin (Rif^S^, Km^S^).

### Plant assays

Surface sterilized seeds of *G. max*, *M. atropurpureum* and *V. unguiculata* were dispersed on agar plates and incubated to germinate in the dark, at 27°C. Once germinated, seedlings were planted in Magenta jars containing vermiculite [31], and watered using nitrogen free B&D solution [44]. Two to three days after their transfer to Magenta jars, each plantlet was inoculated with 200 µl of a solution of 2×10^8^ bacteria. For competition assays, *V. unguiculata* plantlets were inoculated with either 1/100, 50/50 or 100/1 titters of the NGR234/USDA257 or NGR234/G49 combinations. All plants were grown 6 to 8 weeks post inoculation at a day temperature of 27°C, a night temperature of 20°C and a light phase of 12 hours.

### Sample preparation for MALDI-TOF MS identification of free-living bacteria

Prior to spotting MALDI steel target plates, bacteria of G49, NGR234 or USDA257 were prepared using either of the three protocols: (i) direct smear method using a disposable loop, (ii) suspension in 25% formic acid, and (iii) extraction with acetonitrile (ACN) and formic acid (70%). Then, bacterial samples were spotted twenty times on MALDI steel target plates after growth periods at 28°C of 2, 3, 5, 7, 10 and 14 days on plates. Spots were overlaid with 1 µl of matrix consisting of a saturated solution of alpha-cyano-4-hydroxycinnamic acid (Sigma-Aldrich, Buchs, Switzerland) in 33% acetonitrile (Sigma-Aldrich), 33% ethanol and supplemented with 3% trifluoroacetic acid, and air-dried within minutes at room temperature.

### Preparation of nodule bacteria

Once harvested, nodules were frozen and kept at −60°C until further use. Prior to isolation of endosymbiotic bacteria, nodules were surface sterilized in 70% ethanol and crushed into 400 µl of sterile ddH_2_O. For replica plating of nodule bacteria, 20 µl of these homogenates were transferred into independent wells of 96 MicroWell Plates Round Bottom of Nunc (Nunc GmbH & Co. KG, Langenselbold, Germany), and aliquots were subsequently plated on selective media. The rest of the crude supernatants were transferred free of nodule debris into new microfuge tubes and were centrifuged at 20,000 g to collect bacteroids. The bacterial pellets were washed three times with 200 µl of sterile ddH_2_O to remove plant leghemoglobin. Bacteroids were prepared for MALDI-TOF MS fingerprinting as described above.

### MALDI-TOF fingerprinting

Protein mass fingerprints were obtained using a MALDI-TOF Mass Spectrometry Axima™ Confidence machine (Shimadzu-Biotech Corp., Kyoto, Japan), with detection in the linear positive mode at a laser frequency of 50 Hz and within a mass range from 2,000–20,000 Da. Acceleration voltage was 20 kV, and the extraction delay time was 200 ns. A minimum of 10 laser shots per sample was used to generate each ion spectrum. For each bacterial sample, a total of 50 protein mass fingerprints were averaged and processed using the Launchpad™ 2.8 software (Shimadzu-Biotech Corp., Kyoto, Japan). For peak processing of raw spectra, the following settings were selected for Launchpad™ 2.8: (i) the advanced scenario from the Parent Peak Cleanup menu, (ii) a peak width set at 80 chans, (iii) the smoothing filter width set at 50 chans, (iv) a baseline filter width of 500 chans and (v) a threshold apex method set as dynamic. The Threshold offset was set at 0.020 mV with a threshold response factor of 1.2. Each target plate was first externally calibrated using spectra of the reference strain *Escherichia coli* DH5α. In addition, the spectra that were acquired to generate the Superspectra were internally calibrated using strain-specific sets of marker masses at mass-to-charge-ratio (*m/z*) of 4990, 5090, 6188, 6848, 7035, 7480, 7840, 8511 and 8950 for *B. japonicum* strain *G49; m/z* 4979, 5153, 7255, 7484, 8484, 8816, 9239, 10007, 10313 and 11333 for *S. fredii* strain NGR234; and *m/z* 4979, 5153, 7240, 7484, 8144, 8484, 8815, 9305, 10034, 10312, and 11334 for *S. fredii* strain USDA257. The processed spectra were exported as ASCII datasets with each peak being characterized by an *m/z* value and a signal intensity.

### Generation of Superspectra and cluster analysis

Fingerprints of protein masses were analyzed with SARAMIS™ (Spectral Archive and Microbial Identification System, AnagnosTec, Potsdam-Golm, Germany). For each preparation, lists of peaks were imported and binary matrix was calculated using the SARAMIS™ Superspectrum tool with an error window of 800 ppm. Average spectra were generated with Excel macros in which noise masses found in less than half of the twenty replicates were eliminated, resulting in 18 representative averaged spectra (for three preparations and 6 cultivation periods) per strain. For each of the three selected strains, a SuperSpectrum™ consisting in a specific pattern of biomarker masses was calculated using the SARAMIS™ Superspectrum tool. For cluster analysis of protein mass fingerprints, binary matrices were generated using the SARAMIS™ Superspectra tool, and exported as text files. Data was imported into the PAST software (Natural History Museum, Oslo University, Norway) and multivariate neighbor joining cluster analysis was performed using the Dice algorithm to calculate distance matrices. Dendograms were drawn using the FigTree v1.3.1 software (http://tree.bio.ed.ac.uk/software/figtree/) and distance matrices formatted as nexus file.

## Supporting Information

Table S1 Selected biomarkers for SuperSpectra of strains G49, NGR234 and USDA257.
(DOCX)
