# Supplementary material for: In Situ Identification of Plant-Invasive Bacteria with MALDI-TOF Mass Spectrometry
Source: PLoS One. 2012 May 17;7(5):e37189. doi: 10.1371/journal.pone.0037189 (PMC3355115; doi:10.1371/journal.pone.0037189)
Supplement: Table S1 — Selected biomarkers for SuperSpectra of strains G49, NGR234 and USDA257. (DOCX) [file pone.0037189.s001.docx]

**Table S1.** Selected biomarkers for SuperSpectra of strains G49, NGR234 and USDA257.

Masses of ribosomal proteins of NGR234 and G49 were calculated using genomic sequences NC_012587.1 (NGR234 chromosome) or NC_004463.1 (chromosome of *B. japonicum* USDA110), respectively. Ribosomal proteins of USDA257 were derived from contigs of shotgun genomic sequences. Calculated masses were occasionally corrected for absence of N-terminal methionine (^a^) and/or methylation (^b^).

| Mass n° | Experimental mass (Da) ±800 ppm | Present (+) or absent (-) in SSp of | | | Assigned as ribosomal protein of [Da] | Predicted mass as |
| --- | --- | --- | --- | --- | --- | --- |
|  |  | G49 | NGR234 | USDA257 |  |  |
| 1 | 3091.5 ± 2.47 | + | - | - | 50S - L33 [6319]^a^ | [*m/z* + 2H]^++^ |
| 2 | 3100.8 ± 2.48 | - | - | + |  |  |
| 3 | 3249.9 ± 2.59 | - | + | - |  |  |
| 4 | 3250.7+2.60 | - | - | + |  |  |
| 5 | 3318.3 ± 2.65 | + | - | - |  |  |
| 6 | 3385.4 ± 2.70 | - | + | - |  |  |
| 7 | 3393.7 ± 2.71 | - | - | + |  |  |
| 8 | 3419.4 ± 2.73 | + | - | - |  |  |
| 9 | 3433.0 ± 2.74 | + | - | - |  |  |
| 10 | 3447.1 ± 2.75 | + | - | - |  |  |
| 11 | 3461.3 ± 2.76 | + | - | - |  |  |
| 12 | 3468.8 ± 2.77 | - | + | - |  |  |
| 13 | 3475.8 ± 2.78 | + | - | - |  |  |
| 14 | 3489.5 ± 2.79 | + | - | - |  |  |
| 15 | 3502.8 ± 2.80 | + | - | - |  |  |
| 16 | 3517.0 ± 2.81 | + | - | - |  |  |
| 17 | 3574.4 ± 2.85 | + | - | - |  |  |
| 18 | 3588.1 ± 2.87 | + | - | - |  |  |
| 19 | 3598.3 ± 2.88 | - | - | + |  |  |
| 20 | 3702.8 ± 2.96 | - | + | - |  |  |
| 21 | 3719.8 ± 2.97 | - | - | + |  |  |
| 22 | 3740.1 ± 2.99 | - | + | + | 50S - L29 [7483] | [*m/z* + 2H]^++^ |
| 23 | 3812.0 ± 3.05 | + | - | - |  |  |
| 24 | 3880.8 ± 3.10 | + | - | - |  |  |
| 25 | 3970.8 ± 3.17 | - | + | - |  |  |
| 26 | 4096.8 ± 3.27 | - | + | - |  |  |
| 27 | 4166.0 ± 3.33 | - | - | + |  |  |
| 28 | 4177.0 ± 3.34 | - | - | + |  |  |
| 29 | 4240.8 ± 3.39 | - | + | + | 30S - S21 [8484] | [*m/z* + 2H]^++^ |
| 30 | 4450.8 ± 3.56 | - | + | - |  |  |
| 31 | 4502.2 ± 3.60 | + | - | - |  |  |
| 32 | 4508.8 ± 3.60 | - | - | + |  |  |
| 33 | 4614.4 ± 3.69 | - | - | + |  |  |
| 34 | 4618.7 ± 3.69 | - | + | - | 30S - S20 [9370]^a^ | [*m/z* + 2H]^++^ |
| 35 | 4652.2 ± 3.72 | - | - | + | 30S - S18 [9436]^a^ | [*m/z* + 2H]^++^ |
| 36 | 4659.2 ± 3.72 | - | + | - |  |  |
| 37 | 4673.1 ± 3.73 | - | + | + |  |  |
| 38 | 4694.6 ± 3.75 | - | + | - |  |  |
| 39 | 4978.7 ± 3.98 | - | + | + | 50S - L36 [4979] | [*m/z* + H]^+^ |
| 40 | 4990.3 ± 3.99 | + | - | - | 50S - L36 [4990] | [*m/z* + H]^+^ |
| 41 | 4996.5 ± 3.99 | - | - | + |  |  |
| 42 | 5001.9 ± 4.00 | - | + | - | 30S - S15 [10138]^a^ | [*m/z* + 2H]^++^ |
| 43 | 5016.1 ± 4.01 | - | + | + |  |  |
| 44 | 5031.8 ± 4.02 | + | - | - | 30S - S15 [10195]^a^ | [*m/z* + 2H]^++^ |
| 45 | 5090.4 ± 4.07 | + | - | - | 50S - L34 [5090] | [*m/z* + H]^+^ |
| 46 | 5153.9 ± 4.12 | - | + | + | 50S - L34 [5153] | [*m/z* + H]^+^ |
| 47 | 5169.6 ± 4.13 | - | - | + |  |  |
| 48 | 5185.7 ± 4.14 | + | - | - |  |  |
| 49 | 5433.6 ± 4.34 | + | - | - |  |  |
| 50 | 5516.1 ± 4.41 | - | + | - |  |  |
| 51 | 5689.8 ± 4.55 | - | - | + |  |  |
| 52 | 5831.3 ± 4.66 | - | - | + |  |  |
| 53 | 6092.6 ± 4.87 | - | - | + |  |  |
| 54 | 6172.1 ± 4.93 | + | - | - |  |  |
| 55 | 6187.8 ± 4.95 | + | - | - | 50S - L33 [6319]^a^ | [*m/z* + H]^+^ |
| 56 | 6203.1 ± 4.96 | + | - | - |  |  |
| 57 | 6228.8 ± 4.98 | - | - | + | 50S - L33 [6346]^a,b^ | [*m/z* + H]^+^ |
| 58 | 6260.4 ± 5.00 | - | + | - | 50S - L33 [6375]^a,b^ | [*m/z* + H]^+^ |
| 59 | 6275.0 ± 5.02 | - | + | + |  |  |
| 60 | 6299.6 ± 5.03 | - | + | - |  |  |
| 61 | 6344.7 ± 5.07 | - | - | + |  |  |
| 62 | 6381.3 ± 5.10 | - | + | - |  |  |
| 63 | 6505.0 ± 5.20 | - | + | + |  |  |
| 64 | 6532.6 ± 5.22 | - | + | - |  |  |
| 65 | 6634.4 ± 5.30 | + | - | - |  |  |
| 66 | 6775.4 ± 5.42 | - | + | - |  |  |
| 67 | 6788.3 ± 5.43 | - | - | + |  |  |
| 68 | 6848.9 ± 5.47 | + | - | - | 50S - L32 [6979]^a^ | [*m/z* + H]^+^ |
| 69 | 6939.6 ± 5.55 | + | - | - |  |  |
| 70 | 6944.0 ± 5.55 | - | + | - |  |  |
| 71 | 6984.6 ± 5.58 | + | - | - |  |  |
| 72 | 7076.5 ± 5.66 | - | + | - |  |  |
| 73 | 7198.3 ± 5.75 | - | - | + |  |  |
| 74 | 7203.2 ± 5.76 | + | - | - |  |  |
| 75 | 7244.3 ± 5.79 | - | - | + | 50S - L35 [7371]^a^ | [*m/z* + H]^+^ |
| 76 | 7253.4 ± 5.80 | - | + | - | 50S - L35 [7386]^a^ | [*m/z* + H]^+^ |
| 77 | 7409.7 ± 5.92 | - | + | - |  |  |
| 78 | 7420.1 ± 5.93 | - | - | + |  |  |
| 79 | 7480.7 ± 5.98 | + | - | - | 50S - L35 [7611 ]^a^ | [*m/z* + H]^+^ |
| 80 | 7483.4 ± 5.98 | - | + | + | 50S - L29 [7483] | [*m/z* + H]^+^ |
| 81 | 7630.0 ± 6.10 | + | - | - |  |  |
| 82 | 7762.8 ± 6.21 | + | - | - |  |  |
| 83 | 7840.2 ± 6.27 | + | - | - | 50S - L29 [7971]^a^ | [*m/z* + H]^+^ |
| 84 | 8044.7 ± 6.43 | - | + | - |  |  |
| 85 | 8074.1 ± 6.45 | - | - | + |  |  |
| 86 | 8143.9 ± 6.51 | - | - | + | 50S - L31 [8144] | [*m/z* + H]^+^ |
| 87 | 8197.4 ± 6.55 | - | + | - |  |  |
| 88 | 8332.1 ± 6.66 | - | - | + |  |  |
| 89 | 8484.0 ± 6.78 | - | + | + | 30S - S21 [8484] | [*m/z* + H]^+^ |
| 90 | 8511.5 ± 6.80 | + | - | - | 50S - L31 [8511] | [*m/z* + H]^+^ |
| 91 | 8816.2 ± 7.05 | - | + | + | 30S - S17 [8947]^a^ | [*m/z* + H]^+^ |
| 92 | 8952.1 ± 7.16 | + | - | - | 30S - S18 [9081]^a^ | [*m/z* + H]^+^ |
| 93 | 9005.5 ± 7.20 | + | - | - |  |  |
| 94 | 9239.8 ± 7.39 | - | + | - | 30S - S20 [9370]^a^ | [*m/z* + H]^+^ |
| 95 | 9306.5 ± 7.44 | - | - | + | 30S - S18 [9436]^a^ | [*m/z* + H]^+^ |
| 96 | 9321.5 ± 7.45 | - | + | - |  |  |
| 97 | 9348.0 ± 7.47 | - | + | + |  |  |
| 98 | 9466.2 ± 7.57 | + | - | - | 30S - S20 [9593]^a^ | [*m/z* + H]^+^ |
| 99 | 9552.2 ± 7.64 | + | - | - | 30S - S17 [9683]^a^ | [*m/z* + H]^+^ |
| 100 | 10007.2 ± 8.00 | - | + | - | 30S - S15 [10138]^a^ | [*m/z* + H]^+^ |
| 101 | 10035.8 ± 8.02 | - | - | + | 30S - S15 [10165]^a^ | [*m/z* + H]^+^ |
| 102 | 10062.8 ± 8.05 | + | - | - | 30S - S15 [10195]^a^ | [*m/z* + H]^+^ |
| 103 | 10313.1 ± 8.25 | - | + | + | 30S - S19 [10443]^a^ | [*m/z* + H]^+^ |
| 104 | 10411.6 ± 8.32 | - | - | + | 50S - L28 [10540]^a^ | [*m/z* + H]^+^ |
| 105 | 10865.7 ± 8.69 | + | - | - |  |  |
| 106 | 11320.4 ± 9.05 | - | - | + | 30S - S14 [11447]^a^ | [*m/z* + H]^+^ |
| 107 | 11332.7 ± 9.06 | - | + | - |  |  |
| 108 | 12529.8 ± 10.02 | - | - | + |  |  |
| Number of biomarkers | | 39 | 40 | 42 |  |  |
